# Possible *EU futures* for CRISPR-edited plants: Little margin for optimism?

**DOI:** 10.3389/fpls.2023.1141455

**Published:** 2023-03-16

**Authors:** Leire Escajedo San-Epifanio, Igor Filibi, Ainhoa Lasa López, Pere Puigdomènech, Javier Uncetabarrenechea Larrabe

**Affiliations:** ^1^Department of Public Law, Historical-Legal Sciences and History of Political Thought, University of the Basque Country, Bilbao, Spain; ^2^Centre for Research in Agricultural Genomics - CRAG, Spanish National Research Center (CSIC) - Institut of Agro-food Research and Technology (IRTA) - Autonomous University of Barcelona (UAB) - University of Barcelona (UB), Barcelona, Spain

**Keywords:** European integration, EU regulatory framework for plant breeding, EU legislation on new genomic techniques, premarket approval of CRISPR-edited plants, EU status of CRISPR-edited plants

## Abstract

This article addresses the scenarios that may be encountered by the first application for pre-market approval of a CRISPR-edited plant in the EU. Two alternative scenarios are considered in the short and medium term. One of these possible EU futures depends on the final drafting and approval of EU legislation on certain New Genomic Techniques, which was started in 2021 and is due to be quite advanced before the next European Parliament elections in 2024. Since the proposed legislation excludes plants with foreign DNA, two different approval processes for CRISPR-edited plants will coexist if the legislation enters into force: one for plants whose genome has been altered, resulting in mutagenesis, cisgenesis and intragenesis; and the second for plants whose alterations result in transgenesis in general. In the event that this legislative process does not succeed, CRISPR-edited plants in the EU could face a regulatory scenario whose foundations were laid in the 1990s: the regulatory framework that applies to GM crops, food and feed. In this review, an *ad hoc* analytical framework has been built that considers in depth the two possible futures for CRISPR-edited plants in the EU. This framework emphasises the way in which the European Union and the Member States (MS), with their respective national interests, have historically shaped the regulatory framework for plant breeding in the EU. On the basis of the analyses carried out on the two possible futures for CRISPR-edited plants and of their potential with respect to plant breeding, the main conclusions are the following. Firstly, that the regulatory review that started in 2021 is not in itself “good enough” for plant breeding and CRISPR-edited plants. Secondly, that compared to its alternative, the regulatory review currently underway contains at least some promising improvements in the short term. Hence, thirdly, in addition to adopting the current regulation, the MS need to continue to work towards a substantial improvement in the legal status of plant breeding in the EU in the medium term.

## 1 Introduction

The Scottish journalist Allan Little is often quoted as saying that in Europe ‘*history is the unseen guest at every table*’ (Little, 2012), irrespective of the agenda. This has been the case since the period immediately following the end of Second World War, when countries that had been using coal and steel to make weapons to kill each other (see list 1) tried to build the European Coal and Steel Community. One of the main objectives of that initiative was clear: “*the solidarity in production thus established will make it plain that any war between France and Germany becomes not merely unthinkable, but materially impossible*” (Schuman, 1950). Of course, preventing war is not the main element that holds the EU together nowadays, but for the countries of the region there is still something inevitable about EU membership. From a geostrategic point of view, countries of the region cannot afford to remain outside the EU. Although it has not been easy, integration offers numerous synergies and economies of scale to MS, as well as a much greater political reach than they would achieve individually. Therefore, France has described the EU as a power multiplier or *multiplicateur de la puissance* (Verluise, 2017).

However, this is not synonymous with the fact that all Member States (hereinafter MS) on all occasions and in all contexts participate in the EU with the same level of conviction and commitment. At least in the short term, some countries tend to accept decisions that entail sacrificing some of their more immediate interests, negotiating trade-offs in areas that have little or nothing to do with each other. The EU is at essence a common market, although this is not its exclusive function. Once the 27 heads of governments of the MS and the European Commission (hereinafter the EC) have established a possible ‘common interest’ in the Council of the EU or *Consilium* (hereinafter EU Council), the EC is responsible for fulfilling commitments and their legislative implementation, whether in the sphere of the Community institutions or in other international scenarios, with a view towards the global market (Bradford, 2020). The role of the European Court of Justice (hereinafter ECJ), is no less relevant: it is the ultimate guarantor of these Community commitments, supervising the correct implementation and application of EU Law within the constitutional framework defined by the EU Treaties.

Seventy-five years after the Declaration of Rome that gave birth to this unique union, the EU is the product of many concrete achievements and numerous lessons learned from complex situations (such as the introduction of a single currency, the failed attempt to draft a federal constitution or, recently, Brexit). The EU’s governance model combines federal, quasi-federal and confederal elements, and it accommodates the institutional and political diversity of the MS while avoiding disruption of the integration process. This results in a complex multi-scale federalism (Filibi, 2013). Admittedly, from time to time analysts speak of integration fatigue, but the EU continues to expand into new areas and generate innovative policies (Lasa López, 2011; Bradford, 2020) and is radically redefining the classical concept of state sovereignty (Uncetabarrenechea and Filibi, 2022). However, history is always present, so MS are frequently forced to rewrite new consensuses on the palimpsest of older agreements, whether they were good or bad, and on a background of prior crises and disagreements.

The need to be mindful of this institutional peculiarity of the European Union, as well as the presence of historical precedents, becomes especially relevant when the object of analysis is a political and regulatory scenario as dysfunctional as the one experienced by European plant biotechnology. Although negotiation of EU regulations for CRISPR-edited plants for the 2020s seems superficially to be a technical issue, there are significant historical roots. It is important to make this context visible. It is possible to group these issues into three main blocks:

a) The first block of issues arises from the peculiarity of integration of agriculture into the EU. The six funding MS (see below list 1) agreed that agriculture could not be left out of the European project, even if there was still no clear consensus on how to realise its integration (McMahon, 2007). Among other things, the MS debated at the time the favouring of national production over third counties and mechanisms to stabilise prices (Tracy, 1993). Nowadays, related concerns are still alive in the European arena, embodied in expressions such as ‘preservation of independence in food access’, as declared by French ministers after the COVID-19 crisis. Those are some of the factors that explain why successive versions of the Common Agricultural Policy (CAP), which is updated every 5 years, have been so contentious. However, in addition to them, we have to consider that the CAP accounted for 66% of the Community Budget in the early 1980s and currently absorbs a third of the bloc’s entire budget. This underlying background is reflected in many decisions that intersect with agriculture. For example, in 2015, when the approval of the agricultural GMO opt-out was being debated (EP Legal Observatory, 2015), the Committee of the Regions warned that the attitudes of some MS in the GMO decision-making process were not so much related to GMOs as to the positions they advocated on the CAP (CoR, 2015).
b) Another block of issues concerning negotiations on CRISPR-edited plants is the chronic disagreement of MS on plant biotechnology in particular. The EU was one of the first regions in the world to adopt a GMO regulation in the 1990s (Escajedo San-Epifanio, 2008; 2010), but its implementation has been very disappointing. In an official document, the EC acknowledged that decision-making in this area has turned out to be “largely the exception to the usual functioning of the EU comitology procedure as a whole” (EC, 2015). In an attempt to address the situation, the European Parliament (hereinafter EP) voted separately in 2015 for the recognition of an opt-out prerogative for GMO cultivation on the one hand, and an opt-out prerogative for GM food and feed trade on the other hand (EC, 2015). The former was adopted as Directive 2015/412; the latter was rejected by a strong majority of the members of the EP (Escajedo San-Epifanio, 2017). The disagreement, however, is not only related “*to issues associated with the safety of GMOs for health or the environment”* (EC, 2015), but is also the result of the intersection of competing views about the management of domestic agricultural interests, the allocation of funds related to agricultural and plant science research, commercial interests, and the management of plant variety rights (80% of them dominated by just three countries: the Netherlands, France and Germany). Moreover, it should be noted that far from the improvement in the first model of GMO decision-making processes they agreed upon in the 1990s, the obsession of some MS to maintain some political room for manoeuvre has incorporated later reforms that would allow de facto blockages of GM crop authorisations (see below 3.2).
c) A third and final set of concerns is the limited way in which mechanisms of democratic political confrontation have been used in the EU to manage the ideological diversity around GMOs. On the one hand, MS have not reflected together on some forms of anti-GMO attitudes and actions that took place in the late 1990s and the 2000s. All democracies face situations of political conflict where (major) effort is needed to redirect political confrontation over conflicting ideas into the legitimate, constitutionally established spaces and channels set up for this purpose. While political disagreement is considered essential and natural to all democracies, violent expressions of disagreement are to be avoided. Nevertheless, the latter was not factored in when the EU faced anti-GMO attitudes and actions in the late 1990s and 2000s. Indeed, some anti-GMO activists in France, the UK, Germany and Switzerland carried out almost synchronously violent actions against crop-research fields whose institutions had been complying rigorously with regulatory standards (Kuntz, 2012; 2020). Further, governments and courts failed to protect those who were complying with the law. Moreover, a debate on ideas has systematically been avoided and some political scientists have found it striking the way that some politicians adopt parts of anti-GMO discourse and suggest that it is a common sentiment of the majority of European public opinion (Shurman and Munro, 2006; Hayes, 2007; Seifert, 2017; Seifert, 2020). As is often the case, excerpts from political opinion lose meaning when placed out of context. Some forms of rejection to GMOs were and continue to be linked to a deep and legitimate rejection of the dominant model of globalisation and its overriding environmental ethics. Yet, without delving into these positions as a whole, some governments have found it easy to adopt and exploit specific allegations of the anti-GMO movement. Finally, the literature also draws attention to the lack of EC reaction to MS that were notoriously non-compliant with the GMO Directive 90/220/EEC (Lee, 2008) and the permissiveness of the EC in face of the *de facto moratorium* (see details below). The perception of disagreement and ambiguity grew even stronger in 1999 when the EU Council, with many other issues on the table, chose to relegate the status of GM crops, food and feed pending a stronger consensus. Selecting the most urgent matters and postponing the work of negotiating others is not unusual in the EU. More than 20 years have passed since the adoption of Directive 2001/18/EC, and in that time the EU has expanded from 15 MS (see list 3) to 27 MS, even with the loss of the UK. However, the difficulty of adequately confronting political tensions remains unresolved, although some studies suggest that public opinion is evolving (Evanega et al., 2022).

According to the EC, the issues raised by MS around GMOs “*reflect national concerns which do not only relate to issues associated with the safety of GMOs for health or the environment*” (EC, 2015) and now that we are facing the regulatory future for CRISPR-edited plants, it is reasonable to ask whether MS are really in a position to commit to a “sufficiently good” legislation. As Robert Schuman said, a united Europe is not built all at once, but “*through concrete achievements that generate de facto solidarity”* (Schuman, 1950). Each collective achievement relies on the expression of a strong political will by the MS (EC, 1993). What kind of political will have the MS expressed regarding CRISPR-edited plants and what achievements might the EU build from it? Capturing and interpreting that political will is one of the objectives of this research. At least two EU futures may emerge for CRISPR-edited plants in the short term (see 2). The first of these futures is dependent on the approval of an *ad hoc regulation* (2.2). The second future for CRISPR-edited plants involves accommodating CRISPR-edited plants within the regulatory framework that was created in 1990 and reformed in 2001 for GM crops, food and feed (2.3). Both future scenarios - including some possible derivations thereof - are discussed in depth in this paper from political and legal perspectives (see sections 3 and 4), with consideration of how they are affected by “historical issues” (see a, b, c above). Methodologically, the research undertaken has involved tracing three decades of EU regulatory and non-regulatory decisions, as well as failed attempts at regulation and disagreements about plant biotechnology. This work has been carried out with a close eye on the institutional particularities of the EU from the origins of the Common Agricultural Policy to the present day, both in its structural and dynamic dimensions, i.e. the geopolitical and historical evolutionary dimensions.

## 2 Two possible near-term regulatory futures for CRISPR-edited plants in the EU

In 2019, the EU Council launched a process aimed at providing a “*clearer, more evidence-based, applicable, proportionate and sufficiently flexible regulatory framework*” for plants obtained by means of New Genomic Techniques (NGTs), in order to “*cope with advances in science and technology in this area*” (Council Decision 2019/1904). The EC commissioned a study on how this task should be undertaken, with April 2021 as a deadline for submission. Given that 2024 is the date set for the next European Parliament elections (and thus the appointment of a new EC with a new political agenda), it was estimated that if the deadlines were met, a revision draft of the regulation is estimated to be ready within this parliamentary term (EC, 2022). The EC met the deadlines and in Autumn 2021 opened a time period to develop specific legislation for NGT-derived plants (2.1). Depending on whether the envisaged reform is achieved, CRISPR-edited plants will have one of the following two futures in the EU (2.2. and 2.3).

### 2.1 New times are here

In their document “Neue Zeiten, Neue Antworten” – New Times, New Answers- the German Greens (Bündnis 90/Die Grünen, 2018), in contrast to earlier times, reflected on the advisability of analysing whether certain new biotechnologies could help to meet the challenges posed by climate change. Similarly, many other political, scientific and/or governmental voices at European or national levels have recently alluded to the contributions that new genomic techniques could make to the ambitious sustainability goals of the European Green Deal (COM 2018/640), the Farm to Fork Strategy (COM 2020/381) and the Long-Term vision for rural areas (COM 2021/345). In addition, some governments are investing in scientific research programmes in the belief that an agreement will be reached sooner rather than later.

Nonetheless, a political context favourable to an agreement does not always guarantee a good consensus. The starting point of the EU’s plant biotechnology policy was a period with many factors that were conducive to a good agreement. It was the late 1980s and the MS of the (then) EEC (see list 2) were still in the process of completing their internal market. Among the concerns on the table, the White Paper entitled *Completing the internal market* (EC, 1985) stressed the necessity of leaving behind the economic crisis of 1973. Integration required, among other things, a closer approximation of strategic regulations, more agility and efficiency in decision-making processes and a strengthening of the socio-economic cohesion of the region. A goal of strengthening the scientific foundations of the economic community also took on special relevance (EC, 1985). This period of integration coincided with the socio-political emergence of biotechnology. The European institutions launched at that time specific research programs to stimulate the development of biotechnology, as manifested in the Biotechnology Action Program (1985-1989) and later initiatives (e.g. BRIDGE).

As described by the OECD, since 1980 biotechnology has evolved “*from a scientific curiosity towards commercial applications*” (OECD, 1999) and, as a consequence, regulation at the national and international levels of some of its aspects was considered imperative. In the European region, the MS of the then EEC took an important decision at that time because there was a lack of national-level policies on the use of biotechnology, so they embarked together on this new and common regulatory path. Three decades later, we can affirm that the result of that decision has both light and shadows, depending on the biotechnology sector in question. This common path has yielded very good results in some biotechnology sectors -especially the health sector-, but European agri-biotechnologies collapsed due to a regulatory framework with many shortcomings (Escajedo San-Epifanio, 2017).

It is difficult to know whether the outcome would have been different for agri-biotechnologies if the MS had agreed on harmonisation in two stages: a first stage of national regulatory frameworks in the 1990s, followed by a second stage as a prelude to harmonisation at the EU level. We are not aware of any discussion at that time on the possibility of each MS regulating agri-biotechnologies separately, as an alternative to an EU framework. However, it is known that between 2010 and 2015 the possibility of some devolution of agricultural competence with respect to GMOs was discussed (Escajedo San-Epifanio, 2017). Although it did not go as far as a devolution, Directive 2015/412/EC, which recognises an opt-out prerogative for MS (see **Box 1**), can be read along these lines. Given the difficulty of reaching tangible agreements on GMOs, and as a more efficient alternative to an opt-out prerogative, some experts discussed why it would have been preferable to regulate an opt-in prerogative rather than an opt-out prerogative (Escajedo San-Epifanio et al., 2019; Eriksson et al., 2020; 2021). That is, some kind of mechanism that would protect the rights of those MS that wish to use specific GMOs once those GMO crops had been favourably assessed at the EU level.

**BOX 1 Evolution of MS’ policy discretion in GMO decision-making (1990-2015).**

The decision to create a common regulatory framework for GMOs emerged from consensus by the MS in the late 1980s. At that time, there were no previous state frameworks in the MS on this issue, so harmonisation was not necessary. The immediate task was the creation ex novo of a minimum set of rules on the commercial and agricultural use of GMOs, which was expected to move towards further convergence in the future. The tendency to start by agreeing on minimum standards and gradually move towards greater policy convergence has been a constant in many areas of European integration. This is nothing new. But it is striking that, in the specific case of GMOs, there has been a drift over time towards a less united EU. Thus, while scientific assessment and decision-making have been progressively centralised, at the same time there has been a progressive increase in the margin of political discretion surrounding decision-making within the MS.

In the 1990s, the first regulatory framework for GMO crops opted for a system characterised by two elements: decentralisation of scientific assessment complemented by mutual recognition, and final administrative decision-making at the EU level with little room for individual political decision making. Since 2001, important changes have taken place in this respect. On the one hand, there has been centralisation of scientific assessment in the European Food Safety Authority (EFSA), created in 2002. On the other hand, two important actions of political discretion have been granted to the MS -see list 3-: a veto power incorporated in Directive 2001/18/EC, and an opt-out prerogative enacted in Directive 2015/412 (Escajedo San-Epifanio, 2017; González-Vaqué, 2017). These margins of discretion have important implications for the decision-making system (see **Box 4**) and its blocking facility (3.3.1).

The current situation is not comparable to that of the 1980s: The 27 MS know that all possible futures for CRISPR-edited plants should be *EU futures*; a devolution of powers to each of the MS seems to be unthinkable. Additionally, the foundation stone of any regulatory future for CRISPR-edited plants must be placed now, before the 2024 elections to the EP. At this point in time in early 2023, there are only two possible options: the approval of some legal text in the context of the legislative initiative on NGTs (2.2) or the postponement of the decision to the next parliamentary period, which would place CRISPR-edited plants in a *by-default* legal situation until at least 2025 or 2026 (2.3). Let us now compare the main elements of these two possible EU futures concerning CRISPR-edited plants in the EU, leaving the more detailed development of both for Sections 3 and 4.

### 2.2 First possible future: Specific legislation for plants obtained by means of CRISPR?

As indicated in the introduction, the most promising future for CRISPR-edited plants is linked to the legislative process launched by the EC in Autumn 2021. That is the so-called initiative for the creation of legislation for plants produced by certain new genomic techniques, including their food and feed products (EC, 2022). This initiative has already passed the institutional and stakeholder feedback period, as well as the public consultation period.

Since the end of the consultations, there have been three EU actions that point to the possibility of the new regulation being adopted. The first action was the publication of the Factual Summary Report, which indicated that the results of the consultation had been “quite favourable” to the legislative amendment. Secondly, the preparatory work being carried out by EFSA on possible risk assessment criteria for plants derived from targeted mutagenesis and cisgenesis was made public in October 2022 (EFSA, 2022). Finally, as recently as November 2022, the preparation of this legislation on NGTs appeared on the list of tasks that the current EC, led by Ursula von der Leyen, plans to complete before the next European elections.

In section 4 we will examine in more detail what is currently known about the legislation that is in the pipeline. Prior to that, however, let us look at what the future might hold for CRISPR-edited plants if the legislative process does not succeed (2.3).

### 2.3 Second possible future for CRISPR-edited plants: A future by default

Rational choice theory highlighted at least two major explanatory factors that are often overlooked in political analysis (Huntington, 1997): first, that politicians are endlessly opportunistic; and second, that all decisions take place in some type of institutional setting. These factors must also be weighed when the choice made by policy-makers is a decision “to do nothing”. In particular circumstances, doing nothing could be considered a good option or at least an option (Cantekin, 2016), and there are many cases in which policymakers opt for this choice if they consider that for some reason a dispute may be intractable. Applied to the case of CRISPR-edited plants, a hypothetical failure of the EU to reach a consensus in 2023-2024 on plants derived from certain NTGs cannot be interpreted as a lack of awareness of the potential of these techniques for current European plant breeding. To some extent, the legislative failure would be an unfortunate consequence of MS being unable to take on the effort of adopting the new legislation.

What would be the situation for CRISPR-edited plants if the legislation currently being drafted is not adopted? Applying Article 2.2 of Directive 2001/18/EC, CRISPR techniques give rise to GMOs (see details in 2.3.1). Therefore, by default, the same legal status applies to them as to transgenic plants with regard to market access or food use, irrespective of whether the CRISPR technique has led to mutagenesis, cisgenesis, intragenesis or transgenesis. Consequently, the default pre-market approval that will remain for CRISPR-edited plants is composed of two elements:

- As far as agricultural use is concerned, Directive 2001/18/EC on the deliberate release of GMOs would apply.
- In the case that CRISPR-edited plants or products obtained from them were to be used as food or feed, Regulations 1829 and 1830/2003 would also apply. These regulations establish the premarket approval procedure for food uses of GMOs, as well as their labelling and traceability requirements.

Regarding the possible application of this regulatory framework to CRISPR-edited plants, we must make two observations: 1) the particular political use that this regulation makes of some scientific concepts (2.3.1); 2) the veiled criticism that the EU Council has made of this regulation, indicating in 2019 (see above) that NGTs need a *more proportionate, applicable and science-based regulation* (2.3.2).

#### 2.3.1 An observation on the dividing lines used by this old regulatory framework

The establishment of any regulation usually has among its components a section delimiting the scope of the regulation. This is a material decision about the substance of a regulation, but it also needs to be expressed in the text. In the particular case of the EU’s GMO regulatory framework of the 1990s and the 2000s, delimitation was necessary for determining which GM crops and food fall under the EU’s legal capacity and which do not. In order to carry out this delimitation in a very synthetic way, the MS visualised an imaginary subdivision of plant varieties, granting them a different legal status depending on the technique used to obtain them. Accordingly, as we will see below, the EU included a description of dividing lines that separate some subgroups from others as part of the Directive on the deliberate release of GMOs (Directive 220/90/EEC). Let us see how this was done in the legal texts (listed in **Box 1**) applicable to this case.

As far as GM crops are concerned, Directive 220/90/EEC was created primarily with consideration of techniques involving the use of rDNAs, but in the absence of more specific prior legislation, the following was done: firstly, the concept of GMOs in general was defined in the text of the Directive; and secondly, it was specified to which GMOs the legislation applied and which were exempted. Thus, a GMO was defined as any “*organism, with the exception of human beings, in which the genetic material has been altered in a way that does not occur naturally by mating and/or natural recombination*” (art.2.2.). Additionally, a list was included in the annexes defining which techniques were exempted from the provisions of the Directive even though such techniques give rise to GMOs (e.g. classical mutagenesis). Directive 2001/18/EC maintained this approach for the delimitation of GMOs that were subject to and exempted from its application. Consequently, this defined three types of pre-market approval applicable to crops, depending on whether a new plant variety can firstly, be considered GMO or not; and secondly, whether the breeding technique used is an exempted technique or a technique under Directive 2001/18/EC (see summary in **Box 2**).

In other words, a rigorous scientific assessment could conclude that the risk associated with a particular GM food or feed would be equivalent to that of a conventional counterpart (e.g. a food or feed derived from a mutant plant). However, GM food and feed were removed from the novel food category in the 2000s, when specific regulations for genetically modified food and feed were enacted (see above **Box 1**). As some parts of the literature argue, from that point onwards, GM crops, food and feed were given a legal status in the EU that recognises them as distinct from their parent crops (Davison and Ammann, 2017). See summary in **Box 2**.

**BOX 2 Classification of plant varieties according to the premarket approval process foreseen for crops, and the food and feed derived from them in the EU.**

Applying the provisions of Directive 2001/18/EC and other rules on pre-market approval of new plant varieties and their food uses, we can distinguish three groups of plant varieties:

1) Plant varieties that cannot be considered GMOs according to Directive 2001/18/EC, and to which, consequently, Directive 2001/18/EC does not apply. The Regulation. EU 2015/2283 on novel food would only apply in the case that their derived products can be considered novel food.
2) Plant varieties that are GMOs but do not require premarket approval due to the techniques used for their breeding as defined by Directive 2001/18/EC. Their use as food and feed may require authorisation in the context of the EU 2015/2283 Regulation on novel food.
3) Plant varieties that are GMOs and whose premarket approval is subject to the above-mentioned Directive 2001/18/EC. Where applicable, food and feed obtained from these varieties require authorisation under Regulation 1829/2003, as well as compliance with the labelling and traceability rules provided for in Regulation 1830/2003.

#### 2.3.2 A “not sufficiently evidence-based nor proportionate” regulation

The default position of GMO rules applying to CRISPR-edited plants is not good news. As widely recognised, decision-making on GMOs has been stalled for decades in the EU and it is highly doubtful whether any operator would venture to process a pre-market approval application for CRISPR-crops under the current regulatory scenario. At most, stakeholders would only consider submitting applications for authorisation of CRISPR feed crops that possess high commercial interest, but little else would be judged viable. This situation is similar to the experience with GMOs. According to data published by the EC, the absence of GM crops and food in the EU contrasts with the situation for GM feed: soybeans used in animal feed come from countries where more than 90% of soybean production is GM (COM 2015, 176 final). European feed producers cannot compete on a level playing field.

Projecting this situation to the case at hand, this second possible future would be an EU without CRISPR crops and food, but with CRISPR feed imported from third countries and presumably difficulties in correctly identifying them as such (see below). This does not seem to be the situation desired by MS. Indeed, when in the aforementioned 2019 decision the EU Council called on the EC for “more evidence-based, enforceable and proportionate” regulation (Council, 2019), a veiled criticism of the current regulatory framework was revealed. Although not stated directly, the EU Council’s opinion is in line with those expressed by major European scientific institutions, such as ALLEA/EUSAGE, EASAC, Leopoldina or COSCE. All of them suggested that the emergence of NGTs seems a good opportunity to comprehensively review (and improve) the pre-existing plant breeding framework.

The reference to scientific evidence and the idea of proportionality point to the legal need to properly situate NGT-derived plants in relation to the whole range of breeding techniques. A holistic regulatory framework, i.e. one that does not pre-classify risks according to the techniques used (see 2.3.1), would allow MS to control more effectively and proportionately the actual risks of each plant whose genome has been modified. Thus, the report prepared for the STOA (Custers and Dima, 2022: 13) states that new genome editing techniques are “highly precise”, and that in relative terms their risks and uncertainties are “lower than the risks and uncertainties of conventional random mutagenesis, which makes use of radiation or chemicals to induce genetic changes” (Custers and Dima, 2022: 16). However, the current regulation of GMOs, which by default includes CRISPR-engineered plants, does not analyse the risks in this holistic framework. On the contrary, it pre-classifies potential risks according to the technique used to obtain the modified plant and, consequently, a plant obtained by random mutagenesis is considered to be of lower risk than a plant modified by CRISPR, regardless of the specific modification that has been carried out. In fact, only the latter technique is currently subject to authorisation requirements prior to its deliberate release for cultivation (see **Box 3**).

**BOX 3 The advantages of a holistic encompassing regulatory framework: the example of random and targeted mutagenesis.**

Gamma rays are the most widely used mutagenic radiation in plant mutation breeding (Li et al., 2019), and ethyl methanesulfonate or EMA is among the most widely used chemical mutagens. These are two examples of classical mutagenesis. The first results of mutation induction in crop plants were published in the late 1920s and their use became widespread from the 1960s onwards. At the time of GMO regulation in the 1990s, the MS declared the following: first, that mutants obtained by radiation or chemical mutagens are GMOs; second, that pre-market approval is not required for their use in agriculture because they were considered to have a sufficient history of safe use. In contrast to what has been agreed at the EU level, French national legislation states, that random mutagenesis "*does not give rise to GMOs*" (art. L-531, Code de l'environnement). Today, it is possible that in some cases food and feed derived from mutant plants may require authorisation under the Novel Food Regulation 2015 as novel food; but these products are not subject to traceability or labelling procedures comparable to those that apply to GM food and feed (see **Box 2**). Nevertheless, in this future-by-default, a CRISPR-mutated plant would be treated as a transgenic.

This situation does not seem consistent with the comparative sequencing results of conventionally mutated and CRISPR-mutated plants. Today's technology, which did not exist in the 1960s, allows us to test case-by-case impacts on genomes, and it would make sense to have a regulatory framework in which risk assessment focuses on the actual changes made to genomes, rather than being biased or pre-classified by groups of techniques.

We agree with those who have taken a position on the desirability of a holistic review of the regulatory framework for plant breeding (see above), although such a legislative option is not currently on the table. In other words, if the legislative process described in point 2.2 does not succeed, the default future for CRISPR-edited plants will be exactly the same as the one in which transgenic plants have existed since the adoption of Directive 2001/18/EC. However, the implementation of this rule will not be easy. Under current regulations, pre-market approval for agricultural or food use of genome-edited plants sourced from the EU will go through the processes mentioned in **Box 2**. But in the case of genome-edited plants and their products arriving from third countries, the EU is likely to encounter problems in enforcing the labelling and traceability requirements that Regulation 1830/2003 imposes on genetically modified food and feed (Van der Meer et. al., 2023). The information necessary to implement or develop an appropriate detection method for product identification may not be readily available to EU authorities (Ribarits et al., 2021).

## 3 The second possible future for CRISPR-edited plants: “frozen” since 2001

With regard to this regulatory framework-by-default for CRISPR-edited plants, it is also important to note that the legislative approach employed seemed to have “frozen” the framework at the time when Directive 2001/18/EC entered into force. Such a ‘freeze’ would imply, as AG Bobek describes (AG Bobek, 2018) in his opinion on case C-528/16, that twenty years later we would be required to interpret Directive 2001/18/EC on the basis of the *‘factual or social circumstances prevailing when that rule was adopted’*. AG Bobek, as detailed in **Box 4**, argued why such a frozen interpretation should not be accepted. However, the ECJ in its judgment in this case C-528/16, held that in the specific case of GMOs there are reasons that justify limiting the interpretation to the date of adoption of the Directive, which is an exception to the established path of European case law.

At the end of the 1980s, the then MS were facing the socio-economic effects of the 1973 crisis and the previously mentioned White Paper of 1985 encouraged them to speed up the process of completing the internal market. Since strengthening the scientific and technological bases of the economic community was identified as one of the pillars of this process, it was unthinkable that biotechnology policy would remain excluded from this new European era. But building a good regulatory framework for agri-biotechnologies was not an easy task. Moreover, the EU did not yet have a sufficiently solid institutional structure or experience. By contrast, in the US, representatives from more than 18 federal agencies and executive offices spent two years working on the design of a specific and coordinated regulatory framework for agri-biotechnologies. In 1986, the federal government published the Coordinated Framework for the Regulation of Biotechnology (Farquhar and Meyer, 2007), which, as far as crops, food and feed are concerned, has relied on the coordinated action of the FDA, USDA and EPA (US Government, 1986).

The US will not need to pre-classify plant breeding techniques in the same terms as the EEC (vid supra), because the competence over plant breeding, whether GMO or non-GM crops, will be the responsibility of a single institutional structure as a wholeIn the EU, on the other hand, a whole narrative will be built on the aforementioned dividing lines. In essence, the dividing lines that were created to establish who has the legislative capacity for each plant variety (see above) will condition the future of the regulatory framework. This is because the dividing lines embodied two immovable premises. The first premise is that plant breeding techniques, including those yet to come, have been ordered chronologically, and from the simplicity of the discourse it seems that any new plant breeding technology is bound to have more impact and potentially more risk than the pre-existing ones. The second premise, in line with this, is that the European regulatory framework does not need a clause or mechanism to revise its approach in the light of scientific and technological developments.

In an interesting non-binding opinion on case C-528/16, AG Bobek (see **Box 4**) explained in 2018 that the principles of European Community law prevented the GMO regulation from being considered frozen in 2001. It is also interesting to note that the discussion on whether and to what extent NBTs result in GMOs (or a similar regulatory term) has been ongoing since the mid-2000s in many jurisdictions around the world, as detailed in the 2021 paper by Van der Meer et al. However, the ECJ in its judgment on case C-528/16 ruled the opposite way. The ruling did not explicitly refer to whether the interpretation of Directive 2001/18/EC should be understood as “frozen” at its date of creation. Nevertheless, the ECJ did advocate an originalist interpretation or an interpretation in line with the time of the creation of the legislation. Namely, the ECJ stated that the consensus of the MS on certain elements of Directive 2001/18/EC had not been renewed since its drafting and, on that basis, argued that the precautionary principle recommends an interpretation of the Directive in line with its date of creationSee it detailed in **Box 4**.

**BOX 4 A scientific-technical regulatory framework that became ‘frozen’ in 2001.**

In the mid-2010s there was some debate in the EU, given that the wording of Directive 2001/18/EC indicates that "mutagenesis", without further precision, gives rise to GMOs that are exempt from the application of the Directive (see **Box 2**). A court case initiated in France led to a request to the ECJ to clarify whether targeted mutagenesis should (or should not) be considered as an exempted technique, in application of the literal provision of the Directive. AG Bobek, in his non-binding conclusion (AG Bobek, 2018), and the ECJ (ECJ, 2018) adopted a different position on the issue (Casacuberta & Puigdomènech, 2018).

AG Bobek said that while the EU does not have to act on all matters for which it has competence, once the MS decide to legislate on something, there is a constitutional obligation to keep that legislation up to date. Given that some aspects of Directive 2001/18/EC were reformed in 2008 and 2015, AG Bobek said there seems to be no basis for considering that what was not reformed in 2008 and 2015 was frozen in 2001. He also expressed his opinion on the legal argument that New Plant Breeding Techniques resulting in mutagenesis should be understood to be covered by the exemption that the Directive has provided with regard to mutagenesis, as long as they do not involve the use of rDNA. Consistent with that understanding, the AG interpreted the exemption that Directive 2001/18/EC applies to "mutagenesis techniques not involving the use of rDNA" (see **Box 2**) as follows. Any plant obtained by mutagenesis, whether classical or directed mutagenesis, shall be understood to be covered by the regulatory exemption as long as it does not involve the use of rDNA (Bobek, 2018: para 60 and 62; Purnhagen & Wesseler, 2018: 18).

The ECJ, however, made a particular use of self-restraint, as it has been especially criticised for its poor scientific basis (Casacuberta and Puigdomènech, 2018; Escajedo San-Epifanio, 2022). According to the court, mutant plants had been declared exempt from the GMO Directive in 2001 because of their long safety record, and after that date there had been no declaration on plants obtained by directed mutagenesis. For that reason, and applying the precautionary principle, the court considered that it was more appropriate to apply the transgenic regime to edited genome plants than to other mutant plants. However, this understanding ignores the fact that targeted mutagenesis did exist in 2015, the date of the last reform of the Directive. At the same time, it seems that the court recognizes the concept of "long history of safety" as a new criterion or dividing line; that is, as an additional criterion to the dividing lines that the Directive uses to distinguish between GMOs exempted by and subject to its regulatory regime. It so happens, however, that this term (long safety use) does not appear in the text. Moreover, there is no record that the MS reached any explicit agreement (regulatory or political) during the drafting of the standard that specified what is to be understood by the term "long safety record".

### 3.1 The *evolution* of this frozen regulatory framework

Freezing, as is well known, is a preservation technique that, among other things, protects certain objects from the effects of the passage of time. Symbolically, it is used in this case to refer to the regulatory framework for GMOs, because the framework seems to have remained impervious to the passage of time. Why then are we now talking about the evolution of this regulatory framework? It is the case that while the content of the framework will not be revised except in specific areas, the practical application or, as it were, the way the regulatory framework functions will be affected by the passage of time.

The evolution of this fragmented regulatory model over time, together with some problematic elements it already contained from the beginning, will significantly affect the development of agri-biotechnologies in the EU, and because of the freeze, will be an obstacle to adequately accommodate the legal treatment of new plant breeding techniques (Abbot, 2015; Callaway, 2018). Former EU Ombudsman Diamandouros (Diamandouros, 2018) explained in 2018 that although the matter to be regulated was the use of a scientific technological advance, some other policy issues made the regulation of GMOs a “deeply political matter” that needed to be addressed at political level and could not be exclusively confined to an administrative ruling. This is not so much an attribute of the technical content, but is related to the unsatisfactory European decision-making structure for plant biotechnology and the fact that this structure has been in place since the 1990s (Daviter, 2012).

We argue that there are at least three deep policy issues, all of them strategic, that had to be addressed at the political level in the late 1980s, when the EEC was designing what would become Directive 90/220/EEC on the deliberate release of GMOs: the choice of the type of regulatory framework; the specification of GMOs under and exempted from Directive 2001/18/EC; and the decision-making model for authorisation.

The decisions on the regulatory approach (by sectoral or horizontal uses), as well as the delimitation of the EU’s sphere of competence (see 2.3.1) were also decisions with an important political dimension. It is understood that we face a political use of scientific terms beyond the technical delimitation. The dividing line, described in detail in 2.3.1, determined in the early 1990s which GMOs were to be “EEC business”, regulated on the basis of a common consensus, and which were not. And the very existence of this dividing line, which was kept in Directive 2001/18/EC, would also have two important consequences. First, any decision on the dividing line (to leave a future GMO technique to one side or the other) would inevitably have a political component associated with it. In other words, effectively it would mean determining the pre-market approval of a technique, but also defining which level of decision-making (Community or national) would be responsible for taking decisions. The second consequence would be a tendency to reinforce this dividing line, given that the MS would only maintain an important sovereign decision-making capacity over GMOs exempted from the Directive on deliberate release (currently Directive 2001/18/EC).

These decisions predictably led to another question: whether the EEC would use a product-based or process-based evaluation process (Sprink et al., 2016). Since the EEC would not have a holistic competence on plant breeding in general or even on GMOs as a whole (transgenic or not), a product-based evaluation model was very difficult to organise institutionally. Not impossible, but difficult. Nevertheless, it is not conclusively clear that Directive 2001/18/EC opted for a purely process- based regulation (Tagliabue, 2017). In fact, it has been discussed among different institutions and in the literature whether the interpretation and application has been focussed on the resulting GMO organisms, the techniques used to obtain them or a combination of both (Van der Meer et al., 2023).

Let us now look at a key element of the regulatory framework that the EU applies to GMOs under the Directive: the decision-making process.

### 3.2 The decision-making model or *Achilles’ heel* of the EU regulatory framework to be applied by default to CRISPR-edited plants

The decision-making process on the premarket approval of GM crops food and feed has always been the Achilles’ heel of the EU regulatory framework for GMOs. A two-fold need for authorisation decisions had to be addressed when the EEC first regulated the deliberate release of GMOs in the 1990’s. Firstly, there was a need to articulate at the European level some way to address the scientific assessment of risks associated with GM food and feed. Unlike the FDA in the US, created in 1906, The European Food Safety Authority (EFSA) did not exist in the 90s. Secondly, an appropriate panel or committee had to be selected or established to make the final decision on the authorization of a GMO in the light of the scientific assessment.

The initially established model provided, firstly, for a scientific evaluation that could be carried out in any MS under common criteria and, secondly, for final decision-making involving all the MS at the level of the EEC (see **Box 1**). The model initially established provided for a scientific evaluation that could be carried out in any MS under common criteria, and subsequently a final decision-making process involving all the MS at the EEC level. Several factors, however, mostly of a political nature, hindered the proper functioning of this scheme from the outset (see **Box 1**). Between 1998 and 2004, no applications for authorisation of GMOs under Directive 90/220/EC reached the end of the decision-making process (Lee, 2008, 2-3), and some of the 15 MS (see list 3) introduced measures into their national legal systems that aimed to prohibit or hinder national market access for GMOs that had already been authorised at the EU level.

This situation was called “*de facto moratorium*” (Liebermann and Gray, 2006) and was formalised in 1999, when the MS expressed that since a major revision of the regulatory framework was underway - leading to the creation of Directive 2001/18/EC - it did not make sense to continue making authorisation decisions. Nevertheless, the new Directive and the creation of EFSA (in 2002) were not sufficient to unblock the situation. According to the new regulation, when EFSA issues an opinion in favour of the authorisation of a GM crop, the EC prepares a favourable draft decision. The MS then meet and analyse the draft; a favourable draft means that the application meets all the requirements set out in the regulations. The MS have then the political possibility to vote for or against the draft. However, in the event that the MS do not issue any decision, after a period of time, the regulations stated that the EC must formalise the draft decision. Nevertheless, in the few applications that were subsequently processed after 2002, the MS systematically opted for an extraordinary combination of decisions. A non-decision was agreed in the committee (see **Box 1**), but then external pressure was exerted on the EC not to formalise the draft for authorisation. One of the most striking cases was the one that reached the ECJ as case T-164/10, Pioneer Hi Breed Int. (Escajedo San-Epifanio, 2017). In view of the EFSA report, the Commission prepared an authorisation draft in which the MS expressed a non-decision. Subsequently, both the MS and the European Parliament pressured the Commission, recommending it not to formalise the authorisation of the crop. Without mentioning the attitude of the MS and the Parliament’s resolution, the CJEU judgment in the Pioneer case condemned the Commission for its inaction, reminding it that it was obliged to comply with its obligations under Directive 2001/18/EC.

Uncomfortable with this situation, the Barroso Commission initiated a process to increase the discretion of the MS in exchange for the decision to be taken on a more scientific basis - see above **Box 1** -; an arrangement that, as we have already seen, has been widely criticised (Paskalev, 2012). This gave rise to the opt-out prerogative, which allows any MS to partially withdraw from something that other MS have decided at the EU level. While there have been major disagreements in other EU areas, there is no other area in the EU where a similar prerogative is allowed (Salvi, 2016; Poli, 2015) and it has been considered that the *opt out* seems to be more of a trade-off and temporary solution than a long-term solution (Escajedo San-Epifanio, 2017).

## 4 The first (and only truly) possible *EU future* for CRISPR-edited plants: A new regulation

Let us consider the information available to date on the legislation that is being drafted on plants obtained by means of certain NGTs. The main novelty is the creation of a fourth subgroup of plants within the pre-market approval categories of crops, food and feed seen in **Box 2** (see **Box 5**).

**BOX 5 Placing plants obtained by means of NGTs between GMOs exempted from the Directive and GMOs “under Directive”.**

In addition to the status applicable to conventional plants (see **Box 2**), three different statuses for genetically modified plants will coexist in the EU if the current legislation is approved: (1) plants modified by techniques included in the list of exempted techniques in Directive 2001/18/EC; (2) plants obtained by techniques that remain under Directive 2001/18/EC; (3) and plants that, if applicable, would fall under the new legislation on plants obtained by means of certain NGTs.

Regarding food and feed: (a) the GM food and feed regulations would apply to the agri-food use of plants in group 2; (b) Regulation 2015/2883 on novel food would apply to exempted plants (group 1) if they give rise to novel foods; and (c) it remains to be seen what new regulations are envisaged for group 3. Some of the elements of the Roadmap and the fact that DG Health is in charge of proposing the Draft (EC, 2021 IIA) suggest that there will be a specific regulation on the food safety assessment of food and feed derived from plants obtained by NGTs.

Seen from the political perspective and the EC’s effort to generate a consensus among the MS on plants obtained through NGTs, the decision to reference its legal framework with respect to GM plants and those obtained through classical mutagenesis has a certain logic. When complex issues intersect, it is common for international agreements to apply what is known as the technique of compartmentalisation and specialisation of decisions. Narrowing and specifying the margins of agreements tends to allow existing consensus to flourish, thus avoiding the lack of consensus on related issues. In this sense, compartmentalisation can be positive, but it may also have disadvantages, which, as will be seen below, seem to have occurred in this case.

Before going into this, it is worth referring to the legislative instrument and the DG in charge of developing it. The regulation of GM plants is currently contained in different instruments, most notably Directive 2001/18/EC and Regulations 1829 and 1830 of 2003. The Directives allow MS a certain margin of self-regulation when transposing them into their respective legal systems, but this is not the case with the Regulations, which are applied homogeneously throughout the EU. At the current stage of the development of legislation on plants obtained *via* NGTs, it is unknown whether plants obtained through certain NGTs would be regulated by a Directive or a Regulation, but everything suggests that the legislative instrument will be only one of these two possible instruments. In any case, considering that food is involved, it is likely to be a Regulation. The political leadership in drafting the legislation seems to have been entrusted to DG Health, without prejudice to the possibility of coordinating with other bodies on certain elements.

Let us now see how the decision has been compartmentalised and specified in this case. Within the roadmap (EC, 2021, IIA), plants derived from certain NGTS have already been described as requiring regulation with reference to existing legislation (i.e. Directive 2001/18/EC and Regulations 1829 and 1830/2003). Since this reference regulatory framework established groups of organisms, drawing dividing lines according to their breeding processes (2.3.1), it was foreseeable that the technique would also serve as a reference for the delimitation between this prior regulation and the one applicable to NGTS. And, of course, some reference should be made to previous EU experience in assessing: 1) genetically modified plants (as mutants are not assessed *stricto sensu* at the EU level); 2) risks associated with food and feed derived from genetically modified plants (referred to in the legislation as genetically modified food and feed); and 3) risks of food and feed derived from mutants falling within the scope of the novel food legislation (see **Box 2**).

However, the extent to which these inescapable references should or should not limit the freedom of legislators to define an appropriate *ad hoc* framework for GM-derived plants is open to debate. The MS and stakeholders are currently reflecting on this issue. Two things seem to have been neglected from the outset: 1) the scientific-technical assessment of plants derived from certain NTNs would not be the same as that of mutated plants, because only food risks are assessed for the latter and not agricultural risks (see **Box 2**); 2) nor would the assessment of crops, food and feed obtained in this way have the status of transgenic. But this way of thinking, which has always been referenced with regard to mutant and transgenic plants, is guiding the legislative process towards the common assumption that the associated risks of an edited plant must necessarily be presumed to be higher than those of a mutant plant and lower than those of a transgenic plant.

Along these lines, the EFSA published a document in October 2022 entitled “*Criteria for risk assessment of plants produced by targeted mutagenesis, cisgenesis and intragenesis*”, at the request of the EC (EFSA, 2022). In this proposal, it is assumed that “*where the history of safe use of genomically edited plants cannot be sufficiently demonstrated, the function and structure of the introduced allele should be carefully assessed*” (EFSA, 2022), in an approach that suggests the following elements. Firstly, the way in which the regulation establishes the concept of “history of safe use” (hereafter HoSU) will be key, not only in its definition, but especially in terms of allowing the evolution of scientific knowledge to be a reference for adjusting its concrete application. Otherwise, this regulation will remain the same as the GMO regulations: frozen in relation to the time of its formulation. Secondly, it seems that in the case of plants without HoSU, there is a tendency to apply a risk assessment system very similar to the one established for transgenics in Directive 2001/18/EC. This is despite the fact that both the report submitted to the STOA in 2022 (Custers and Dima, 2022) and EFSA itself (EFSA, 2022) have acknowledged that, in some cases, plants obtained by directed mutagenesis or cisgenesis do not pose new risks compared to those produced by mutant plants or even those obtained by conventional breeding techniques.

There are some inconsistencies associated with the above-mentioned EU Council objective of providing a “*clearer, evidence-based, enforceable, proportionate and sufficiently flexible regulatory framework*” for plants obtained by new genomic techniques, in order to “*cope with advances in science and technology in this field”* (Council Decision 2019/1904).

It seems that we are unlikely to achieve these objectives in this way. In terms of clarity, it does not seem a good decision to place such importance in a concept like HoSU, which is a legally indeterminate concept. Nor does it seem to achieve the objective of proportionality, given the difference between plants obtained through NGTs on the one hand, and mutants and conventionally bred plants on the other. For the latter, HoSU is not required to decide on their agricultural use, and at the same time, *ad hoc* assessment will only be used in relation to their food use if it is considered that substantial equivalence cannot be determined with respect to the homologous (conventional or classical mutants). However, contrary to the scientific opinions provided by the EFSA on NGT-derived plants, the possibility that some of them can be considered as equivalent to conventional or mutant plants in terms of agricultural or food use seems to be generally excluded.

These difficulties, however, are not only due to the criteria proposed for scientific assessment, but are the consequence of very different governance models for the risks associated with crops, food and feed in the EU and the US. The current institutional model in the EU is far from providing the regulatory flexibility that exists in the US through the FDA, USDA and environmental authorities, acting separately and/or jointly.

## 5 Conclusion: A milestone in the history of EU plant breeding is approaching, but it is not the end of the journey

The years 2021 to 2024 will be reported in the political history of European plant breeding as the first time the EU tried to provide CRISPR-edited plants with a regulatory framework, but it will not be the last. Of the two possible futures that have been described, only one of them is somewhat promising for CRISPR-edited plants, but only somewhat. In our opinion, a legislation whose risk assessment gives more weight to obtained products and their specific uses than to processes alone would be closer to the objectives expressed by the EU Council, namely, to achieve a “*clearer, evidence-based, enforceable, proportionate and sufficiently flexible regulatory framework*”.

Nevertheless, the legislation in the pipeline *remains promising* because it seems that the EU has come to this point with some lessons learned. The GMO regulatory space has proven to be dysfunctional in many respects and the MS today continue to express conflicting views. History, as an invisible guest, is very much present in this scenario, and as explained above, a holistic review of all rules applying to plant breeding would have been unthinkable. As an alternative, the EC has made a very important effort to put on the table what should be on the table and exclude what should not. The goal has been to avoid competing interests that, while part of the long journey from farm to fork, can only be extrinsically related to plant breeding decisions. Seen in comparison to how the regulatory framework for GMOs has evolved, the merit of the EC in finding its own space for genome-edited plants should not be minimised.

This short-term future is, however, only promising to a certain extent, because in our opinion the proposed legislation will not be the best possible, even if it is passed. In terms of comparison with the other possible futures (or future *by default*, see above 2 and 3) it is certainly an acceptable option. But it should not be overlooked that in itself, the legislation underway pays the price of being overly referenced to or constrained by the influence of the GMO regulatory framework. This being the case, it is virtually impossible to shield the regulation of genome edited plants from the deep issues that have surrounded GMOs (see 1, especially a, b and c). This is particularly evident in the approach to risk assessment (see 5), although it is likely to be felt in other aspects of regulation.

The best approach for this specific legislation would have been to act as if there were a holistic framework for plant breeding and as if we had to fit genome-edited plants into that puzzle. In that way, flaws in other pieces of the puzzle, in particular transgenics, would not have unduly influenced this legislative development.

In any case, with the end of the current parliamentary term imminent, it is unlikely that the approach will be redirected. We must therefore start working now for the post-2024 future. Let us hope that the MS do not close this legislation but that work continues in the medium term to ensure a regulatory framework for European plant breeding that, while adequately protecting the environment and human health, offers plant breeders legal certainty to remain and develop in the EU.

## 6 Legal provisions cited in the text

Directive (EU) 2015/412 of the European Parliament and of the Council amending Directive 2001/18/EC as regards the possibility for the MS to restrict or prohibit the cultivation of genetically modified organisms (GMOs) in their territory, OJ L 68, 13.3.2015, p. 1–8.

Directive 2001/18/EC of the European Parliament and of the Council on the deliberate release into the environment of genetically modified organisms. Current consolidated version, after EU Regulation 2019/1381 of 20 June 2019: 27/03/2021.

Regulation (EC) No 1829/2003 of the European Parliament and of the Council on genetically modified food and feed, OJ L 268, 18.10.2003, p. 1–23.

Regulation (EC) No 1830/2003 of the European Parliament and of the Council concerning the traceability and labelling of genetically modified organisms and the traceability of food and feed products produced from genetically modified: Official Journal L 268, 18/10/2003.

Regulation (EU) 2015/2283 of the European Parliament and of the Council on novel foods: OJ L 327, 11.12.2015, p. 1–22.

## 7 EU MS lists, by date

List 1. EEC founding Members. France, West Germany, Italy, the Netherlands, Belgium and Luxembourg.
List 2. EEC members, late 1980s: Belgium, Denmark, France, Germany, Greece, Ireland, Italy, Luxembourg, Netherlands, Portugal, Spain and United Kingdom.
List 3. EU members 2001. Austria, Belgium, Denmark, Finland, France, Germany, Greece, Ireland, Italy, Luxembourg, Netherlands, Portugal, Spain, Sweden, and the United Kingdom.

## Data availability statement

The original contributions presented in the study are included in the article/supplementary material. Further inquiries can be directed to the corresponding author.

## Author contributions

LE, IF, AL, and JU conceived and designed the study and the structure of the manuscript, as well as the basis for each of its sections. LE wrote the first draft of the manuscript. LE, IF, AL, and JU wrote sections of the advanced manuscript. PP reviewed in particular the technical sections of the manuscript. All authors contributed to the article and approved the submitted version.
